# Screening of unusual forms of diabetes might not have been accounted by the Brazilian public health system

**DOI:** 10.1186/1758-5996-7-S1-A194

**Published:** 2015-11-11

**Authors:** Ricardo Emidio Navarrete de Toledo, Wimbler Pires Silva, Laura Priscila N Toledo, Maryane Caroline de Toledo, Rebeca Esteves Matos Rodrigues Ramiro, Bruna Maria Grosso Mascarenhas, Adriana Seixas Costalonga, Julio Cesar Rebelo Sampaio Filho, Ana Julia Garcia Pereira, Andrea Abbud, Giovanna Mura, Julyana Pereira Navajas, Ana Teresa Mana Gonçalves Santomauro, Fadlo Fraige Filho

**Affiliations:** 1 Beneficencia Portuguesa de São Paulo, São Paulo, Brazil

## Background

Type 2 diabetes is among the major public health problems of the 21st century and is associated with an alarming rise in the incidence of obesity, spreading fast among youngsters. In adults, it accounts for about 90 to 95 percent of all diagnosed cases of diabetes. However, even being more prevalent, type 2 diabetes is not the only possibility for slim people in this age group.

## Objective

The present study aimed to discuss the correct diagnosis in non-obese patients aged 20-39 yrs. classified as having type 2 diabetes mellitus (T2D).

## Materials and methods

Epidemiological study based on data obtained from Brazilian System of Registration and Accompaniment of Hypertensive and Diabetic Patients (http://hiperdia.datasus.gov.br). All of the cases diagnosed as type 2 diabetes in 2013-2014 were divided into two age groups and separated into two another 2 groups (obese and non-obese). For statistical analysis, we used the age groups between 20-39 yrs. Data refers to patients monitored by the Program of Health of the Family.

## Results

We evaluated 15,468 patients, of whom 7944 were in the obese group and 7524 in the non-obese group.

## Conclusion

**Figure 1:**
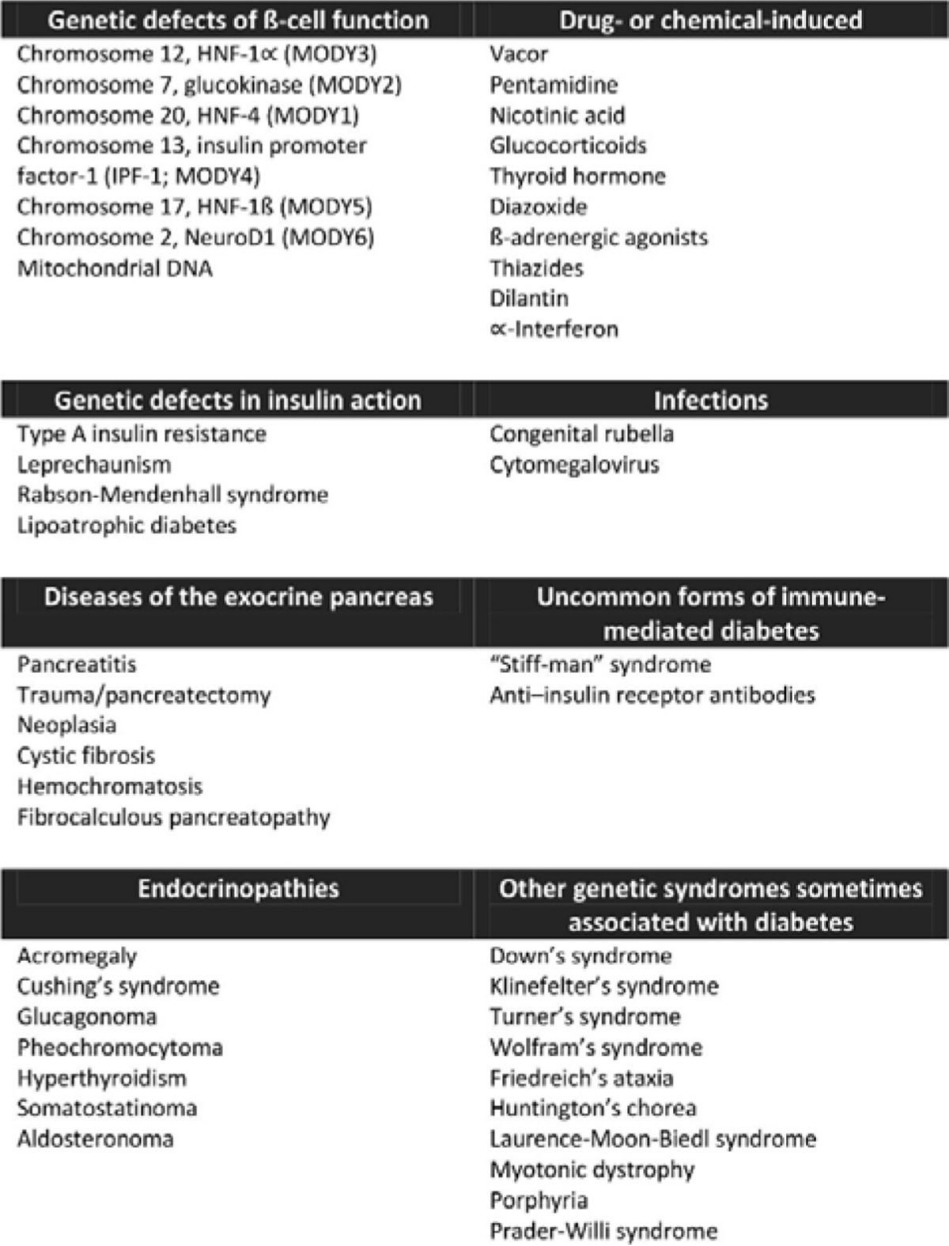
Other specific types of diabetes mellitus.

“Other specific types of diabetes” is a heterogeneous category that refers to unusual forms of diabetes. Traditional examples resulting from specific genetic syndromes (also known as Maturity Onset Diabetes of the Young or MODY), cystic fibrosis, autoimmunity, malnutrition, infections, hemochromatosis, surgical causes and drug causes (Figure 1). Altogether they account for 1-2% of all diagnosed cases of diabetes. Even more important than these uncommon types of diabetes, is the necessary medical knowledge in order to recognize it. Rates of type 2 diabetes are increasing dramatically in Latin American. Currently there is no way to explain the unexpectedly high rates of diabetes in nonobese individuals, which lead many experts to think that these less common forms might be masked as well. To be certain, challenges remain that should not be underestimated. Although all these forms of diabetes identified until now has been responsible for a small fraction in diabetic population, surely will provide in the future the basis in which rests the heterogeneity of diabetes and its long-term complications. More than that: it can also suggest that both autoimmune insulitis and insulin resistance may coexist in parallel and autoimmunity precipitates the onset of hyperglycemia.

